# Short-Term Effect of the Inclusion of Silage Artichoke By-Products in Diets of Dairy Goats on Milk Quality

**DOI:** 10.3390/ani10020339

**Published:** 2020-02-21

**Authors:** Paula Monllor, Gema Romero, Esther Sendra, Alberto Stanislao Atzori, José Ramón Díaz

**Affiliations:** 1 Departamento de Tecnología Agroalimentaria, Universidad Miguel Hernández de Elche, 03312 Alicante, Spain; pmonllor@umh.es (P.M.); esther.sendra@umh.es (E.S.); jr.diaz@umh.es (J.R.D.); 2 Dipartimento di Agraria, Sezione di Scienze Zootecniche, Università degli studi di Sassari, 07100 Sassari, Italy; asatzori@uniss.it

**Keywords:** silage, lipid profile, minerals, metabolic profile

## Abstract

**Simple Summary:**

The use of artichoke by-products, both from the canning industry or from the stubble that remains in the field, provides a cheaper source of nutrients, suitable for ruminant feeding due to their ability to digest fibre-rich foods. The use of these by-products for animal feed is also a way to reduce waste caused by the canning industry and disposal costs, as well as the area and resources allocated to the production of food for livestock, contributing to the circular economy. Evaluating the effect of the inclusion of silage artichoke by-products (bracts and rest of crop plant) in dairy goat rations on the milk yield and composition, animal health status, mineral and lipid profile is an effective way to explore the suitability of these alternative feedstuffs for goat cattle. The use of artichoke bracts and whole plant silage in dairy goat diets does not lead to marked differences in the milk yield and quality or the animals’ health status. From a nutritional point of view for human health, slightly better mineral and lipid profiles are observed in milk from goats fed artichoke plant silage, due to its higher polyunsaturated fatty acids and conjugated linoleic acid contents.

**Abstract:**

Artichoke by-products represent a high amount of waste whose removal entails several costs. Moreover, feed is the main cost in a farm. So, including these by-products in ruminant diets would lower feed costs. Two experiments were conducted to evaluate the effect of two levels of inclusion, 12.5% and 25.0%, of two silages of artichoke by-products (artichoke bracts, AB and artichoke plant, AP) in the diet of goats on the milk yield, composition and quality and on the metabolic profile of the animals. AB presented the lowest blood urea content and there were no differences in milk yield in the two experiments. However, with 25.0% of silage by-product in the diet, a higher fat content was observed in AB and of protein in AP, as well as this treatment showing a slightly higher Se content. Regarding the milk lipid profile, milk from 12.5% of AP treatment presented a higher PUFA content. In conclusion, the use of silage artichoke by-products in dairy goat diets does not jeopardise milk yield and quality and health status of animals and, from a nutritional point of view for human health, a slightly better mineral and lipid profile is observed in milk from AP treatments.

## 1. Introduction

The use of agri-food by-products and alternative fodder provides local food for livestock and it helps to reduce dependence on food from abroad. The use of vegetable by-products, both from the canning industry or from the stubble that remains in the field, is a cheaper source of nutrients, suitable for ruminant feeding due to their ability to digest fibre-rich foods. The use of these by-products for animal feed is also a way to reduce waste caused by the canning industry and disposal costs, as well as the area and resources allocated to the production of food for livestock, contributing to the circular economy.

The marked seasonality of vegetables reduces the availability of these foods for much of the year and their high water content makes them a food with a short shelf life. Previous studies have shown that the silages of these by-products meet the fermentative conditions that ensure the nutritional and safety quality necessary to be part of the ration of small ruminants [1,2,3], and it allows their conservation for long periods of time (up to 200 days, according to [3]). The references found in the literature on the effect of silage consumption of these by-products in sheep on the milk quality and composition and the health status of the animals are scarce, but indicate their suitability for this purpose [1,4,5,6]. However, there has only been one study conducted in dairy goats on the effect on the technological properties of milk [7].

The consumption of fresh goat’s milk worldwide is in third place behind that of cow and buffalo [8], but demand for it is increasing as a source of animal protein, calcium and phosphorus [9], and it has been catalogued by medical professionals as a perfect substitute for cow’s milk in cases of food allergies [10].

The artichoke (*Cynara scolymus* L.) yield worldwide is important and generates a large quantity of by-products. In 2017, 1,505,328 t of artichoke were harvested worldwide [11]. This crop contributes significantly to the agricultural economy of the Mediterranean, where more than 60% of the world production of this vegetable originates [12]. Overall, artichoke by-products from the canning industry (leaves, external bracts and stems) represent a high amount of waste material—about 80% of the total biomass of the plant [13]—which entails the generation of 1,204,262 t/year of by-product. The artichoke plant is a by-product composed of leaves, stems and some unharvested inflorescences, which has traditionally been used for small grazing ruminants or has been harvested and taken to dairy farms [1]. According to Wernli and Thames [14], the yield of green fodder in this crop is 11.1 t/ha; which, taking into account the area cultivated worldwide (122,390 ha; [1]), results in a production of 1,358,529 t/year of available artichoke plant.

This study aims to evaluate the effect of the inclusion of silage artichoke by-products (bracts and the rest of the crop plant) at two levels (12.5 and 25.0% on a dry basis) in the ration of dairy goats on the milk yield and composition, animal health status, milk mineral and lipid profile and indices related to the nutritional quality of milk fat. The hypothesis of this study is that the inclusion of these by-product silages in well-balanced total mixed rations does not jeopardise goat performance and health status.

## 2. Materials and Methods

### 2.1. Animals and Facilities

Lactating Murciano-Granadina goats were used, housed at the teaching and experimental farm of the Miguel Hernández University, with a straw bed, access to outdoor yards, free access to water and enough feeding space for all animals (at least 35 cm/animal). The animals were fed twice a day, at 8:00 a.m. and 2:00 p.m., and milked once a day (Casse milking parlour, 2 × 12 × 12, GEA, Germany), as usual in the region. This study was approved by Responsible Research Office from Miguel Hernández University (code UMH.DTA.GRM.01.14).

### 2.2. Experimental Design

From a group of 70 goats that were in the middle of lactation (fourth month) fed with a conventional diet (control, C), pre-experimental sampling was performed and 57 animals were selected, with an average body weight of 44.7 ± 6.84 kg, an average production of 2.68 ± 0.68 kg/day and a somatic cell count (SCC) of 5.97 ± 0.42 Log cells/mL. They were divided into three homogeneous groups according to the variables mentioned (without significant differences between treatments). Each group was randomly assigned a diet: C (control, no by-products), AB (with silage artichoke bracts), AP (with silage artichoke plant).

The C diet was a conventional ration that included alfalfa hay and a mixture of grains in both experiments and was similar to that of the pre-experimental period. In Experiment 1, the level of inclusion of by-products in AP and AB was 12.5% (on dry matter basis), while in Experiment 2 the level of inclusion of silage by-products was increased to 25.0% (on dry matter basis) of the total ration, and the rest of the ration was composed of alfalfa hay and grain mixture. The groups of both experiments were composed of the same animals. All rations were calculated according to the formulation recommendations of Fernández et al. [15] for goats with a milk yield of 2.5 kg/day in Experiment 1 and 2.0 kg/day in Experiment 2, so that they were isoenergetic and isoproteic and adjusted to the milk yield. The animals were fed twice a day with fixed amounts, not ad libitum. Table 1 shows the amounts of the ingredients in each diet, as well as their composition and the amount offered daily.

After the pre-experimental sampling was carried out, Experiment 1 began, which lasted 8 w. The first 2 w served for each group of animals to adapt to their treatment diet (with 12.5% silage by-product). In the next 6 w, 4 biweekly samplings were performed. Next, Experiment 2 began. After a 4-week adaptation period to the diets that included 25.0% by-product, three biweekly samplings were performed in the next 4 w.

### 2.3. Variables Analysed

Representative samples of each ration were taken at the beginning of each experiment for subsequent laboratory analysis. The composition of the rations (Table 1) was determined by the AOAC [17] methods for dry matter (DM, g/kg; method 930.5), organic matter (OM, g/kg DM; method 942.05), ether extract (EE, g/kg DM; method 920.39) and crude protein (CP, g/kg DM; method 984.13). The neutral detergent fibre (NDF, g/kg DM), acid detergent fibre (ADF, g/kg DM) and acid detergent lignin (ADL, g/kg DM) contents were analysed according to Van Soest et al. [18]. The total polyphenol content (TP, g/kg DM) was analysed by the Folin-Ciocalteu method described in Kim et al. [19]. The apparent in vitro dry matter digestibility (IVDMD, g/kg DM) was analysed by the method of Menke and Steingass [20]. The proportion of short chain volatile fatty acids (VFA, g/kg DM): acetic, propionic and butyric acid, including also lactic acid and ethanol were determined by liquid chromatography (HPLC 1200 Agilent, Santa Clara, CA, USA) and Supelcogel C-610H column: 30 cm × 7.8 mm ID, (Saint Louis, MO, USA); [21]).

The analysis of the fatty acid profile in the diets was performed by direct methylation on the lyophilised samples, without prior extraction of the fat, according to Kramer et al. [22]. Fatty acid methylated esters (FAME) were identified by a gas chromatograph (GC-17A Shimadzu, Kioto, Japan) coupled to a flame ionisation detector (FID) equipped with a capillary column (DB23 30 m × 0.25 mm ID × 0.25 µm film coating JW Scientific, Agilent, USA). A mixture of FAME (18912-1AMP, Sigma-Aldrich, Saint Louis, MO, USA) was used for the identification of the fatty acids present in the samples.

For the analysis of dietary and milk minerals, a previous digestion of the samples was carried out according to González-Arrojo et al. [23]. Na, Mg, K, Ca, P, S, Se, Zn, Cu, Fe and Mn were determined by a quadrupole ICP-MS chromatograph (Agilent, Santa Clara, CA, USA) using an internal calibration.

The milk yield of each animal (kg/day) was determined during the milking of each sampling by a Lactocorder^®^ device (Lactocorder, Balgach, Switzerland). The macrocomposition of milk (fat, protein, useful dry matter content, UDM; true protein, casein, whey protein, lactose, dry matter, DM; non-fat dry matter content, NFDM; ash; %) was analysed by medium infrared spectroscopy (MilkoScan™ FT2, Foss, Hillerød, Denmark) and somatic cell count (SCC, 10^3^ × cell/mL) using the electronic fluoro-optical method (DCC, DeLaval, Tumba, Sweden). The fat-corrected milk yield was calculated according to Gravert [24]: FCM (3.5%) = 0.433 × yield (kg/day) + 16.218 × fat yield (kg/day), and fat and protein corrected milk yield according to Schau and Fet [25]: FPCM = yield (kg/day) × (0.337 + 0.116 × Fat (%) + 0.06 × Protein (%). At each sampling, the animals were weighed using a scale with a precision of 100 g (APC, Baxtran, Vilamalla, Spain) to study the evolution of body weight (BW, kg) during the experiment. Food intake was determined by the difference from the amount (in DM basis) that was offered and refused, on two consecutive days in the week of each sampling and determining the DM of a representative sample of ration offered and refused, dried in an oven at 105 °C for 48 h. For the analysis of the fatty acid profile in the milk samples, an extraction was carried out using the Folch method with some variations described in Romeu-Nadal et al. [26] and a subsequent methylation according to the method of Trigueros and Sendra [27]. The chromatograph, column and FAME mix for the identification of milk fatty acids were the same as those used for diets. Indices related to the nutritional quality of milk fat were calculated: Atherogenicity Index (AI) and Thrombogenicity Index (TI) according to Batista et al. [28] and the Desaturase Index (DI) for C14:0, C16:0 and C18:0 according to Lock and Garnsworthy [29].

Milk samplings were carried out during the milking in the weeks in lactation 16 (pre-experimental sampling), 20 and 24 (in Experiment 1) and 28 and 32 (in Experiment 2), blood samples were taken from jugular vein of the fasting animals using an Eclipse™ needle (BD Vacutainer, Franklin Lakes, NJ, USA) and collected in three test tubes with 4 mL capacity (BD Vacutainer, Franklin Lakes, NJ, USA): one of them contained potassium oxalate and NaF for the analysis of glucose, and another contained lithium heparin and was reserved for the analysis of urea, β-hydroxybutyrate (BHB) and haematocrit. Finally, the tube containing EDTAK2 was used to collect blood for the analysis of cholesterol and non-esterified fatty acids (NEFA). Blood samples were analysed by enzymatic spectrophotometry. A glucose oxidase/peroxidase kit (Ref. 11503 and 11505, Biosystems, Barcelona, Spain) was used for glucose and cholesterol (mg/dL), the kinetic method GN 10125 developed by Gernon (Spain) was used for urea (mg/dL), the Ranbut d-3-Hydroxybutyrate kit (RB 1007, Randox, Crumlin, UK) was used for BHB (mmol/L) and an enzymatic-spectrophotometric method (FA 115, Randox, Crumlin, UK) was used for NEFA (mmol/L). The percentage of haematocrit was determined with a microhaematocrit.

### 2.4. Statistical Analyses

The SCC values were transformed into base ten logarithm to carry out the statistical analysis. The data from each experiment (Exp. 1 and Exp. 2) were analysed separately following a mixed linear model with repeated measures PROC GLIMMIX (SAS v9.2, 2012), introducing in the model the covariate of the data obtained in the pre-experimental sampling, for the statistical analysis of Experiment 1, or of the last sampling of Experiment 1 for the analysis of Experiment 2, according to the following equation:

Y = µ + D_i_ + S_j_ + D_i_xS_j_ + covY_0_ + A_k_ + e (1)

where Y is the dependent variable, µ is the intercept, D_i_ is the fixed effect of the diet (i = C, AB, AP), S_j_ is the fixed effect of sampling (j = 1, 2, ...), D_i_xS_j_ is the interaction of the diet with the sampling, covY_0_ is the effect of the value of Y in the pre-experimental sampling, A_k_ is the random effect of the animal and e the residual error. The analysis of the data of the milk mineral and lipid profile did not take into account the effect of sampling or its interaction with the diet effect. A covariance model of composite symmetry was used, as it presented the best modelling of the data (according to the AIC and BIC statistics).

## 3. Results

### 3.1. Body Weight, Milk Yield and Composition and Plasmatic Metabolism

**Figure 1:**
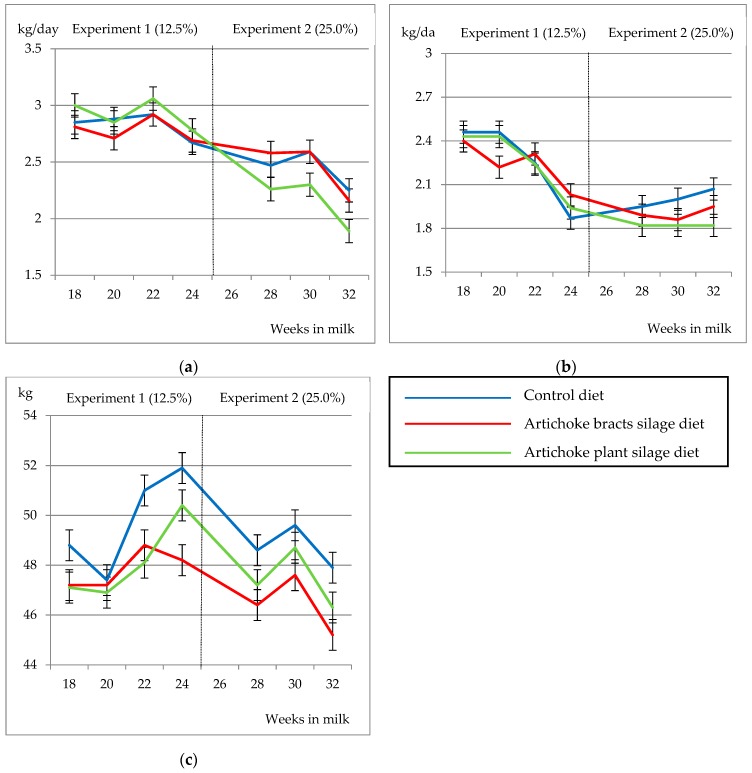
Changes in milk yield (**a**), fat corrected milk (**b**) and average body weight (**c**) due to diet throughout the lactation in experiments 1 and 2.

In Exp. 1, the inclusion of by-products did not cause significant differences in almost any variable (Table 2). Although there were no differences in initial BW (homogeneous groups), and average BW increased by 3.90 kg (*p* < 0.001) during the experiment in the three treatments, the diet had a significant effect on BW, as it increased more in C and AP than in AB (49.8, 48.1 and 47.8 kg for C, AP and AB, respectively). On a daily average, the values of dry matter intake (DMI) for the different treatments were 2.14 ± 0.059, 2.08 ± 0.073 and 2.12 ± 0.076 kg DM/day in C, AB and AP, respectively. Regarding the milk yield and composition, no differences due to diet were observed, but the effect of sampling and interaction diet × sampling were observed. Milk yield, as well as FCM and FPCM yields and feed efficiency, were reduced at the end of the experiment, although FCM yield of AB and feed efficiency of AP remained stable. The ash content also decreased (−0.188%; *p* < 0.001). The concentration of the rest of the variables related to the composition and LSCC increased throughout the experiment with slight differences between treatments, so the interaction was significant. Although the interaction diet × sampling was significative in some of the studied variables, this was due to small fluctuations observed throughout the experiment and they did not cause important differences, as can be seen in Figure 1.

Regarding blood metabolites, the inclusion of 12.5% in the dry matter basis of AB and AP did not change the levels, except in the case of plasma urea, where AB had a lower content than C (43.2 vs. 47.7 mg/dL; *p* < 0.05) and AP obtained an intermediate content (44.6 mg/dL). During the experiment, urea levels increased equally in the three treatments (+4.84 mg/dL; *p* < 0.001) and haematocrit levels in AP (+1.71%; *p* < 0.01), while cholesterol levels were reduced in C (−7.40%; *p* < 0.05) and those of NEFA in AP (−0.201 mmol/L; *p* < 0.05).

In Exp. 2, the effect of diet was significant in many of the variables analysed (Table 3). There were no statistical differences in initial BW, but a reduction in average BW was observed in the three treatments, resulting in a lower BW in AB (46.4 kg; *p* < 0.001). Irrelevant differences in DMI were observed, resulting for AB, C and AP equal to 1.86 ± 0.051, 1.92 ± 0.038 and 1.92 ± 0.052 kg DM/day, respectively. The treatments did not show differences in milk yield and feed efficiency, but in FCM and FPCM yields, which were higher (*p* < 0.05) in C and AB in contrast to AP, without differences between AB and AP in FPCM. The effect of sampling was significant in FCM and FPCM, which were reduced by 0.337 and 0.225 kg/day (*p* < 0.001), respectively, between the beginning and the end of the experiment, and also in feed efficiency related to FPCM. AB was the treatment with the highest fat content in milk (5.35%; *p* < 0.01), as well as in the parameters related to fat, such as UDM (9.62%) and DM (14.4%). These three variables were reduced by 1.39%, 1.40% and 1.31% (*p* < 0.001) during the experiment without any diet × sampling interaction. On the contrary, AP presented a higher concentration in the protein fractions—crude protein (4.36%), true protein (4.01%) and whey protein (0.556%)—as well as in NFDM (9.82%) and in LSCC (5.90 Log10 cell/mL). During the experiment, the casein content was reduced 0.061% (*p* < 0.001) and those of whey protein and lactose increased by 0.048 and 0.100% similarly in all three treatments. However, NFDM and the crude and true protein levels increased only in AB and AP, while the concentration of these two protein fractions was reduced in C. As for LSCC, it only increased in AP (+0.192 Log10 cell/mL; *p* < 0.001).

Regarding the blood metabolite profile, the differences found were small. A slightly higher level of cholesterol was observed in AB (111 mg/dL; *p* < 0.05) and urea in C and AP (43.2 and 42.2 mg/dL, respectively; *p* < 0.01). During the experiment, the glucose and haematocrit levels were reduced (*p* < 0.001), the concentration of cholesterol decreased in AB and NEFA in AP, while BHB increased slightly (*p* < 0.01) in this treatment.

### 3.2. Milk Mineral Profile

Regarding the mineral content of milk (Table 4) in Experiment 1, only Cu was affected by the diets. AP obtained a lower content than C (94 vs. 111 µg/kg; *p* < 0.05), without differences with AB (105 µg/kg). Including 25.0% of the by-products in the diet (Experiment 2), only S, Cu and Se were affected by the diet. It was observed that the Cu level of the two experimental treatments was slightly lower than C (80.6 vs. 95.1 µg/kg; *p* < 0.05). Regarding the content of S, AB was lower than C (365 vs. 405 mg/kg; *p* < 0.05), without differences with AP (402 mg/kg). Similarly, the amount of Se present in AB milk (33.3 µg/kg) was slightly lower than that C and AP (36.6 and 40.7 µg/kg, respectively; *p* < 0.05), without differences between these two treatments.

### 3.3. Milk Lipid Profile

In Experiment 1, the significant differences obtained between treatments were small and can be considered irrelevant from a biological point of view (Table 5). In comparison to C, the inclusion of 12.5% of AB in the ration resulted in a small reduction (*p* < 0.01) of the milk PUFA (polyunsaturated fatty acids) content (−0.58%). AP slightly increased the level of MCFA (medium chain fatty acids) (+0.7%; *p* < 0.05), as the milk from this treatment also had the highest concentrations of myristic (C14:0) and palmitic (C16:0) acid and also a higher DI of C16:0 (+0.006; *p* < 0.05). A slightly lower content of n3 fatty acids and higher n6/n3 ratio (*p* < 0.05) were observed in treatments that included artichoke by-products in Experiment 1. The milk of the animals fed with a 25.0% of AB had a lower level of MUFA (monounsaturated fatty acids) and LCFA (long chain fatty acids) than C (−1.0 and −1.1%, respectively; *p* < 0.05), mainly because they also obtained lower oleic (C18:1 cis9, −1.3%) and arachidonic acid contents (C20:0, −0.130%).

## 4. Discussion

### 4.1. Body Weight, Milk Yield and Composition and Plasmatic Metabolism

The BW values of the three treatments are within the normal range for the Murciano-Granadina breed [30]. The lower BW of AB can be explained by the lower initial BW of this treatment in both experiments, although there were no significant differences in the initial stage. In addition, the AB diet had higher values of ammonia (N), which also has a satiating effect due to the gamma-aminobutyric acid produced in the liver [31] and could have reduce DMI, Huhtanen et al. and Krizsan and Randby [32,33] observed in calves and dairy cows fed with grass silages. FCM and FPCM yields were higher in C and AB in Experiment 2, as C was the treatment with the highest milk yield (although without differences from the other two treatments) and AB had the highest fat content, so the milk of AB had the highest content in UDM and DM too. However, this did not happen in Jaramillo et al. [5], where the inclusion of up to 30% of artichoke silage in sheep diets did not lead to differences in milk composition. All the treatments showed a good level of feed efficiency, which were higher than those observed with other diets in goats of different breeds [34,35,36]. The absence of significant differences in milk yield and feed efficiency ensure the good feed quality of these by-products. The slightly higher protein content in the milk of the experimental treatments (especially AP) observed in Experiment 2 could be due to a higher level of TP in this diet, which would form complexes with the dietary protein, making it less soluble and, therefore, less digestible by ruminal microflora, so that it would increase the digested protein in the small intestine [37]. The decrease in milk yield and the increase in macrocomposition values observed in the three treatments during the experiment are related to the normal progress of lactation. The results for daily milk yield, fat, protein and lactose are slightly higher than those observed by Vacca et al. [38] in Murciano-Granadina goats, while the LSCC values are similar to those found in this study. Therefore, the use of artichoke silage at the levels of inclusion tested does not appear to have detrimental effects on the milk yield or composition, similar to that observed in cow’s milk [39] and sheep [5]. In general, the inclusion of silages in well-balanced diets does not jeopardise the yield and composition of milk, as various authors observed by including different types of by-product silages in small ruminants’ diets. While the inclusion of olive cake silage in sheep and goats’ rations had no effects on milk yield [40,41], a higher fat content was observed in the milk of sheep and goats fed sliced oranges silage [42] and tomato and olive by-product silages [43], as occurred with AB in Experiment 2.The blood metabolite profile is one of the main indicators when evaluating the physiological state of animals [44]. The absence of relevant differences between treatments indicates the viability of the inclusion of these by-products in the ration for Murciano-Granadina dairy goats at the tested doses. The urea values measured in plasma were slightly higher than those of Ibáñez et al. [45] in Murciano-Granadina goats in mid-lactation (30.6 mg/dL), as the protein content of the diets was lower (132 g/kg DM), while the values of BHB and NEFA (1.74 and 1.06 mmol/L, respectively) were lower in our experiment due to lower mobilisation of body reserves, as also shown by the higher plasma glucose content compared to that observed by Ibáñez et al. [45] (43.9 mg/dL). The slight lower urea content of AB can be explained by two causes. On the one hand, the AB diet had a TP content slightly higher than the other two (Table 1). Frutos et al. [46] related the tannins (which are part of the TP) with a reduction in the digestibility of the protein in the rumen, thus releasing less ammonia, and the subsequent urea synthesis in the liver is reduced [47]. Decreases in milk urea were also observed in sheep fed diets with low levels of TP [48,49]. On the other hand, the slightly lower DMI of AB could cause a lower protein metabolism. However, the urea reduction was small and did not translate into lower protein content in milk or in FPCM yields.

### 4.2. Milk Mineral Profile

The milk mineral profile of the three treatments is similar to that reported by Guo [8] in goat milk. While in Experiment 1, the inclusion of 12.5% of AP and AB barely caused differences (only in Cu content), increasing the level to 25.0% caused a slight decrease in Se, as was observed in AB, although such small magnitudes that can be considered irrelevant.

### 4.3. Milk Lipid Profile

The milk lipid profile was not significantly modified by the inclusion of by-products in the diets at the doses tested. The milk lipid profile is strongly influenced by diet [48,49], in addition to the breed of animals. Some differences were observed between the results of this study with those of Ibáñez et al. [45], where they used the same breed: while the palmitic and α-linolenic (C18:3n3) contents were higher (42.6 and 0.400%, respectively), the oleic, linoleic (C18:2n6), conjugated linoleic (CLA) and vaccenic acid (C18:1t11) levels were lower (14.2, 2.46, 0.41 and 0.39%, respectively) than those observed in our experiment, with the exception of AB, which presented a higher level of vaccenic acid. In Mancilla-Leytón et al. [50], these differences were also found in grazing goats in the Mediterranean region, in addition to a higher ratio of SFA/UFA and AI index (3.03 and 3.00, respectively) and lower values in PUFA content (4.47%) and in the C16:0 and C18:0 DI (0.02 and 0.56). All this indicates that the inclusion of artichoke by-products in goat diets improves the milk lipid profile due to the higher content of oleic acid, PUFA and CLA, which have anti-atherogenic properties and reduce obesity [51]. In comparison to the rest of the treatments, the inclusion of 12.5% of AP in the diet showed a slightly higher content of PUFA, so it would have a more cardio-healthy lipid profile. On the other hand, the SFA/UFA ratio is a health indicator of the nutritional value of animal fat in human nutrition [52]. Simopoulos [53] recommends a value below 0.45 for the PUFA/SFA ratio for foods designed to slow cardiovascular diseases and cancer, which has been achieved by all treatments (0.118 in Experiment 1 and 0.113 in Experiment 2) and this means that the consumption of this milk can counteract the effects of diets high in saturated fatty acids, which are common in most western countries.

## 5. Conclusions

The use of silage artichoke bracts and whole plant in dairy goat diets at the doses studied (12.5 and 25.0%) in substitution for other forage sources does not lead to marked differences in the milk yield and quality or in the health status of the animals. From the point of view of nutritional quality of milk for human health, there is a slightly better lipid and mineral profile in AP. Consequently, the inclusion of artichoke bracts and plant silages in well balanced diets will allow us to take advantage of the high by-product availability for ruminant diets without negative effects on animal performance.

## Figures and Tables

**Table 1:** Ingredients and chemical composition of the experimental diets.

Item	Experiment 1			Experiment 2		
	C	AB	AP	C	AB	AP
Ingredients (g/100 g DM)						
Alfalfa hay	37.6	24.3	24.2	37.6	15.0	12.0
Barley straw	-	0.959	-	-	0.632	-
Grains mix	59.2	61.2	59.8	59.2	59.5	56.2
Oat	3.16	1.24	-	3.18	-	-
Soybean meal 44%	-	-	2.63	-	-	5.68
Silage	-	12.4	13.4	-	24.6	25.8
Premix vitamins/minerals	-	-	-	-	0.316	0.352
kg DM offered/day	2.25	2.19	2.24	1.98	1.90	1.99
Chemical composition						
DM (g/kg FM)	872	577	707	868	398	516
g/kg DM						
OM	935	935	930	929	937	922
EE	57.3	58.3	58.1	56.7	60.6	53.8
CP	146	148	148	150	143	144
NDF	432	411	454	452	443	442
ADF	206	203	204	205	200	210
ADL	44.6	41.1	54.6	45.2	42.9	41.0
PT	1.59	2.84	2.04	2.88	4.27	3.55
IVDMD	717	728	717	699	650	687
^1^ ME (Mcal/kg DM)	2.57	2.53	2.55	2.46	2.55	2.48
VFA and fermentation products (g/kg DM)						
Lactate	n.d.	n.d.	11.1	n.d.	n.d.	24.9
Acetate	18.6	24.7	28.7	19.3	24.0	25.1
Propionate	n.d.	n.d.	5.35	n.d.	12.2	n.d.
Butyrate	n.d.	n.d.	n.d.	n.d.	4.60	n.d.
Ethanol	n.d.	3.09	n.d.	n.d.	8.20	n.d.
Ammonia N (g N-NH_3_/kg N_T_)	1.40	9.38	2.65	2.41	20.9	3.48
Fatty acids profile (g/100 g total fatty acids)						
C4:0	0.053	2.30	0.072	0.052	7.31	0.246
C6:0	0.067	0.547	0.068	0.047	1.966	0.151
C12:0	0.244	0.108	0.095	0.123	0.087	0.089
C14:0	0.429	0.359	0.349	0.410	0.358	0.351
C16:0	17.7	16.5	16.8	17.4	16.1	17.9
C16:1c9	0.266	0.389	0.290	0.268	0.297	0.291
C18:0	3.72	3.44	3.38	3.48	3.03	3.59
C18:1c9	25.5	25.7	26.1	24.9	21.3	24.5
C18:1c11	1.03	1.04	1.11	0.99	0.97	1.06
C18:2n6	44.3	44.3	46.4	45.5	41.2	45.5
C18:3n3	3.86	2.80	2.79	3.97	3.45	3.47
C20:0	0.498	0.422	0.497	0.472	0.420	0.486
C20:1n9	0.314	0.314	0.329	0.312	0.297	0.309
C22:0	0.510	0.184	0.483	0.462	0.369	0.411
C24:0	0.211	0.279	0.367	0.392	0.322	0.330
SFA	24.2	24.9	22.5	23.3	32.2	24.1
MUFA	27.3	27.6	28.0	26.6	23.0	26.3
PUFA	48.5	47.6	49.4	50.1	44.8	49.6
Mineral profile						
Na (g/kg DM)	2.02	2.24	4.16	2.57	2.58	4.56
Mg (g/kg DM)	3.14	3.12	3.00	3.13	3.01	2.67
K (g/kg DM)	13.6	14.7	15.1	13.9	15.4	15.5
Ca (g/kg DM)	8.45	7.96	8.33	8.60	6.66	9.12
P (g/kg DM)	2.72	3.33	3.07	3.17	3.31	3.19
S (g/kg DM)	3.17	2.94	3.10	3.10	2.98	2.94
Se (mg/kg DM)	0.243	0.188	0.176	0.375	0.336	0.350
Zn (mg/kg DM)	53.3	54.2	46.3	59.5	73.0	61.4
Cu (mg/kg DM)	6.84	6.68	6.60	6.16	6.50	6.09
Fe (mg/kg DM)	274	351	217	373	277	257
Mn (mg/kg DM)	45.6	54.4	44.8	62.7	61.0	62.5

C: Control diet, AB: Diet with artichoke bracts silage, AP: Diet with artichoke plant silage, DM: Dry matter, FM: Fresh matter, OM: Organic matter, EE: Ether extract, CP: Crude protein, NDF: Neutral detergent fibre, ADF: Acid detergent fibre, ADL: Acid detergent lignin, TP: Total polyphenols, IVDMD: In vitro dry matter digestibility, EM: Metabolic energy, VFA: Volatile fatty acids, SFA: Saturated fatty acids, MUFA: Monounsaturated fatty acids, PUFA: Polyunsaturated fatty acids; 1 [16].

**Table 2:** Results of the comparison of means of the variables related to body weight, food intake, milk yield and composition, somatic cell count and basal metabolism, according to the effects considered in Experiment 1.

Variable	Diet					Sampling Signification	Interaction Signification
	C	AB	AP	SEM	Signification		
Initial BW (kg)	43.7	44.6	45.7	1.44	n.s.	−	−
Average BW (kg)	49.8 a	47.8 b	48.1 a,b	0.62	*	***	***
Milk yield (kg/day)	2.26	2.24	2.26	0.076	n.s.	***	n.s.
FCM (3.5%; kg/day)	2.83	2.78	2.92	0.103	n.s.	***	*
FPCM (kg/day)	2.66	2.61	2.72	0.087	n.s.	***	n.s.
Feed efficiency (Milk yield/DMI)	1.06	1.07	1.07	0.060	n.s.	***	***
Feed efficiency (FPCM/DMI)	1.23	1.27	1.28	0.071	n.s.	***	**
Fat (%)	5.10	5.16	5.48	0.212	n.s.	***	***
UDM (%)	9.41	9.28	9.78	0.255	n.s.	***	***
DM (%)	14.1	14.0	14.4	0.26	n.s.	***	***
NFDM (%)	9.60	9.59	9.56	0.070	n.s.	***	***
Protein (%)	4.23	4.20	4.30	0.064	n.s.	***	***
True protein (%)	3.90	3.90	3.95	0.056	n.s.	***	***
Casein (%)	3.37	3.35	3.40	0.048	n.s.	***	***
Whey protein (%)	0.532	0.518	0.550	0.016	n.s.	***	***
Lactose (%)	4.21	4.23	4.16	0.029	n.s.	***	*
Ash (%)	0.469	0.453	0.478	0.018	n.s.	***	*
LSCC (cell/mL)	5.77	5.81	5.75	0.061	n.s.	**	*
Glucose (mg/dL)	59.2	59.9	60.7	1.16	n.s.	n.s.	n.s.
Cholesterol (mg/dL)	113	117	116	2.4	n.s.	*	*
Urea (mg/dL)	47.7 a	43.2 b	44.6 a,b	1.21	*	***	n.s.
BHB (mmol/L)	0.442	0.400	0.382	0.022	n.s.	n.s.	**
NEFA (mmol/L)	0.463	0.370	0.444	0.047	n.s.	*	*
Haematocrit (%)	29.6	28.9	29.3	0.43	n.s.	***	**

C: Control diet, AB: Diet with artichoke bracts silage, AP: Diet with artichoke plant silage, SEM: Standard error mean; BW: Body weight, DMI: Dry matter intake, FCM: Fat corrected milk, FPCM: Fat and protein corrected milk, UDM: Useful dry matter (% fat + % protein), DM: Dry matter, NFDM: Non-fat dry matter, LSCC: Log_10_ somatic cell count, BHB: β-hydroxybutyrate, NEFA: Non-esterified fatty acids; a, b, c: different letters in the same row indicate significant difference between diets. * *p* < 0.05; ** *p* < 0.01; *** *p* < 0.001.

**Table 3:** Results of the comparison of means of the variables related to body weight, food intake, milk yield and composition, somatic cell count and basal metabolism, according to the effects considered, in Experiment 2.

Variable	Diet					Sampling Signification	Interaction Signification
	C	AB	AP	SEM	Signification		
Initial BW (kg)	50.1	48.6	50.9	0.92	n.s.	−	−
Average BW (kg)	48.7 a	46.4 c	47.4 b	0.36	***	***	n.s.
Milk yield (kg/day)	2.01	1.90	1.82	0.072	n.s.	n.s.	n.s.
FCM (3.5%; kg/day)	2.44 a	2.44 a	2.15 b	0.086	*	***	n.s.
FPCM (kg/day)	2.31 a	2.28 a,b	2.05 b	0.078	*	***	n.s.
Feed efficiency (Milk yield/DMI)	1.02	1.05	0.95	0.058	n.s.	n.s.	n.s.
Feed efficiency (FPCM/DMI)	1.18	1.23	1.08	0.066	n.s.	***	n.s.
Fat (%)	4.88 b	5.35 a	4.73 b	0.149	**	***	n.s.
UDM (%)	9.14 a,b	9.62 a	9.06 b	0.178	*	***	n.s.
DM (%)	13.9 a,b	14.4 a	13.8 b	0.17	*	***	n.s.
NFDM (%)	9.69 b	9.80 a,b	9.82 a	0.046	*	***	**
Protein (%)	4.22 b	4.28 a,b	4.36 a	0.046	*	**	*
True protein (%)	3.89 b	3.94 a,b	4.01 a	0.041	*	**	*
Casein (%)	3.37	3.43	3.45	0.033	n.s.	***	*
Whey protein (%)	0.523 a,b	0.506 b	0.556 a	0.013	**	***	n.s.
Lactose (%)	4.34	4.31	4.30	0.027	n.s.	**	n.s.
Ash (%)	0.441	0.476	0.436	0.015	n.s.	n.s.	n.s.
LSCC (cell/mL)	5.77 a,b	5.72 b	5.90 a	0.061	*	**	***
Glucose (mg/dL)	59.5	57.2	59.8	1.03	n.s.	***	n.s.
Cholesterol (mg/dL)	109 a,b	111 a	102 b	2.5	*	***	***
Urea (mg/dL)	43.2 a	38.9 b	42.2 a	1.00	**	n.s.	n.s.
BHB (mmol/L)	0.410	0.417	0.405	0.019	n.s.	**	**
NEFA (mmol/L)	0.631	0.717	0.625	0.054	n.s.	*	***
Haematocrit (%)	31.6	32.1	32.1	0.55	n.s.	***	**

C: Control diet, AB: Diet with artichoke bracts silage, AP: Diet with artichoke plant silage, SEM: Standard error mean; BW: Body weight, DMI: Dry matter intake, FCM: Fat corrected milk, FPCM: Fat and protein corrected milk, UDM: Useful dry matter (% fat + % protein), DM: Dry matter, NFDM: Non-fat dry matter, LSCC: Log_10_ somatic cell count, BHB: β-hydroxybutyrate, NEFA: Non-esterified fatty acids; a, b, c: different letters in the same row indicate significant difference between diets. * *p* < 0.05; ** *p* < 0.01; *** *p* < 0.001.

**Table 4:** Effect of the diet on milk mineral profile in Experiments 1 and 2.

Variable	Experiment 1					Experiment 2				
	C	AB	AP	SEM	Signification	C	AB	AP	SEM	Signification
Na (mg/kg)	354	373	369	11.7	n.s.	343	350	362	11.6	n.s.
Mg (mg/kg)	160	162	161	7.6	n.s.	167	156	166	3.9	n.s.
P (mg/kg)	1152	1245	1200	62.6	n.s.	1171	1142	1181	41.4	n.s.
S (mg/kg)	408	401	403	16.6	n.s.	405 a	365 b	402 a,b	10.7	*
K (mg/kg)	1492	1513	1560	61.6	n.s.	1405	1415	1514	39.8	n.s.
Ca (mg/kg)	1394	1536	1449	58.9	n.s.	1437	1447	1451	36.8	n.s.
Mn (µg/kg)	64.2	77.2	89.8	13.36	n.s.	70.1	68.9	62.7	5.60	n.s.
Fe (µg/kg)	457	678	499	154.2	n.s.	483	403	468	26.7	n.s.
Cu (µg/kg)	111 a	105 a,b	94 b	4.1	*	95.1 a	80.6 b	80.6 b	3.74	*
Se (µg/kg)	25.8	26.5	26.3	2.93	n.s.	36.6 a,b	33.3 b	40.7 a	1.67	*
Zn (µg/kg)	4811	4734	4968	225.3	n.s.	5387	4925	4987	286.1	n.s.

C: Control diet, AB: Diet with artichoke bracts silage, AP: Diet with artichoke plant silage; SEM: Standard error mean; a, b, c: different letters in the same row indicate significant difference between diets. * *p* < 0.05; ** *p* < 0.01; *** *p* < 0.001.

**Table 5:** Effect of the diet on milk fatty acid profile (g/100 AGT) in Experiments 1 and 2.

Variable	Experiment 1					Experiment 2				
	C	AB	AP	SEM	Signification	C	AB	AP	SEM	Signification
C6:0	1.17	0.94	0.61	0.228	n.s.	0.420	0.945	0.881	0.250	n.s.
C8:0	3.21	3.31	3.33	0.124	n.s.	3.29	3.18	3.36	0.081	n.s.
C10:0	9.08	9.36	9.58	0.228	n.s.	9.07	9.16	9.36	0.177	n.s.
C12:0	5.33	5.50	5.58	0.114	n.s.	5.17	5.27	5.40	0.086	n.s.
C13:0	0.117	0.105	0.104	0.012	n.s.	0.108	0.077	0.104	0.020	n.s.
C14:0	10.1 b	10.3 a,b	10.5 a	0.11	*	10.1	10.1	10.5	0.13	n.s.
C14:1c9	0.070	0.124	0.077	0.018	n.s.	0.153	0.104	0.115	0.036	n.s.
C15:0	1.16	1.03	1.04	0.045	n.s.	1.12	1.24	1.16	0.076	n.s.
C15:1	0.168	0.153	0.146	0.013	n.s.	0.168	0.150	0.159	0.013	n.s.
C16:0	23.0 b	23.5 a,b	23.8 a	0.20	*	24.0	24.3	24.2	0.45	n.s.
C16:1	1.17 b	1.24 a,b	1.35 a	0.052	*	1.22	1.19	1.29	0.034	n.s.
C16:2	1.17	1.17	1.14	0.078	n.s.	0.968	1.133	1.182	0.092	n.s.
C17:1	0.307	0.339	0.333	0.013	n.s.	0.345	0.390	0.363	0.023	n.s.
C18:0	12.3	11.8	11.4	0.32	n.s.	13.2	12.7	12.2	0.34	n.s.
C18:1t11	0.300	1.458	0.067	0.477	n.s.	0.193	1.866	0.106	0.814	n.s.
C18:1c9	22.5	21.7	23.2	0.62	n.s.	22.5 a	19.8 b	22.5 a	0.77	*
C18:2t9,12	1.31 a	0.72 b	1.44 a	0.107	***	1.23	1.69	1.43	0.421	n.s.
C18:2n6	3.40	3.47	3.56	0.063	n.s.	3.28	3.24	3.31	0.062	n.s.
C18:3n6	0.156 a,b	0.176 a	0.142 b	0.010	*	0.161	0.164	0.146	0.042	n.s.
C19:0	0.121	0.118	0.089	0.014	n.s.	0.124	0.099	0.103	0.024	n.s.
C18:3n3	0.302	0.269	0.275	0.013	n.s.	0.309	0.329	0.252	0.032	n.s.
CLA c9t11	1.06	1.07	1.19	0.057	n.s.	0.866	0.674	0.876	0.079	n.s.
CLA t10c12	0.208	0.232	0.145	0.056	n.s.	0.095	0.298	0.151	0.064	n.s.
∑CLA	1.27	1.31	1.33	0.069	n.s.	0.961	0.971	1.027	0.112	n.s.
C20:0	0.382	0.307	0.315	0.030	n.s.	0.374 a	0.244 b	0.307 a,b	0.035	*
C20:1n9	0.160	0.136	0.112	0.037	n.s.	0.100	0.064	0.090	0.022	n.s.
C21:0	0.082 a	0.048 a,b	0.030 b	0.017	*	0.046	0.027	0.047	0.011	n.s.
C20:4n6	0.203	0.190	0.203	0.009	n.s.	0.225	0.211	0.225	0.017	n.s.
C20:5n3	0.131 a	0.093 a,b	0.086 b	0.014	*	0.124	0.116	0.102	0.020	n.s.
C24:1	0.064 a	0.027 b	0.026 b	0.011	*	0.052	0.072	0.029	0.030	n.s.
C22:6n6	0.034 a,b	0.015 b	0.048 a	0.008	*	0.039	0.031	0.033	0.005	n.s.
SFA ^1^	66.1	66.3	66.4	0.34	n.s.	66.9	67.4	67.7	0.41	n.s.
MUFA ^2^	24.7	25.2	25.4	0.33	n.s.	24.6 a,b	23.6 b	24.6 a	0.30	*
PUFA ^3^	7.97 a	7.41 b	8.22 a	0.154	**	7.30	7.88	7.71	0.56	n.s.
UFA ^4^	32.7	32.6	33.6	0.40	n.s.	31.9	31.5	32.3	0.44	n.s.
SFA/UFA	2.02	2.04	1.98	0.034	n.s.	2.10	2.14	2.09	0.042	n.s.
SCFA ^5^	13.5	13.6	13.5	0.25	n.s.	12.8	13.3	13.6	0.37	n.s.
MCFA ^6^	42.6 b	43.4 a,b	44.1 a	0.41	*	43.3	43.9	44.5	0.53	n.s.
LCFA ^7^	42.7	41.9	42.4	0.45	n.s.	42.8 a	41.7 b	41.9 a,b	0.29	*
n3	0.433 a	0.362 b	0.361 b	0.022	*	0.433	0.445	0.353	0.047	n.s.
n6	3.79	3.85	3.95	0.060	n.s.	3.71	3.64	3.72	0.095	n.s.
n6/n3	8.94 b	10.68 a	11.03 a	0.512	*	8.58	8.80	10.74	0.870	n.s.
AI ^8^	2.38	2.39	2.41	0.039	n.s.	2.42	2.52	2.49	0.038	n.s.
TI ^9^	3.14	3.10	3.08	0.042	n.s.	3.28	3.39	3.27	0.063	n.s.
DI C14:0	0.007	0.012	0.007	0.002	n.s.	0.015	0.010	0.011	0.004	n.s.
DI C16:0	0.051 b	0.053 a,b	0.057 a	0.002	*	0.051 a,b	0.049 b	0.053 a	0.001	**
DI C18:0	1.86	1.96	2.05	0.067	n.s.	1.71	1.70	1.85	0.062	n.s.

C: Control diet, AB: Diet with artichoke bracts silage, AP: Diet with artichoke plant silage; SEM: Standard error mean, DI: Desaturation index; ^1^ SFA (saturated fatty acids) = C6:0 + C8:0 + C10:0 + C12:0 + C13:0 + C14:0 + C15:0 + C16:0 + C18:0 + C19:0 + C20:0 + C21:0; ^2^ MUFA (monounsaturated fatty acids) = C14:1c9 + C15:1 + C16:1 + C17:1 + C18:1t11 + C18:1c9 + C20:1n9 + C24:1; ^3^ PUFA (polyunsaturated fatty acids) = C16:2 + C18:2t9,12 + C18:2n6 + C18:3n6 + C18:3n3 + CLAc9t11 + CLA t10c12 + C20:4n6 + C20:5 + C22:6n6; ^4^ UFA (unsaturated fatty acids) = MUFA + PUFA; ^5^ SCFA (short chain fatty acids) = C6:0 − C10:0; ^6^ MCFA (medium chain fatty acids) = C11:0 − C17:0; ^7^ LCFA (long chain fatty acids) = C18:0 − C24:0; ^8^ AI (atherogenic index) = $\frac{C12:0+4\times C14:0+C16:0}{MUFA+n6+n3}$; ^9^ TI (thrombogenic index) = $\frac{C14:0+C16:0+C18:0}{0.5\times MUFA+0.5\times n6+0.3\times n3+n3/n6}$; a, b, c: different letters in the same row indicate significant difference between diets. * *p* < 0.05; ** *p* < 0.01; *** *p* < 0.001.
